# Correction: Habibi I. et al. “Clinical and Genetic Findings of Autosomal Recessive Bestrophinopathy (ARB)” Genes, 2019, *10*, 953

**DOI:** 10.3390/genes11050503

**Published:** 2020-05-03

**Authors:** Imen Habibi, Yosra Falfoul, Margarita G. Todorova, Stefan Wyrsch, Veronika Vaclavik, Maria Helfenstein, Ahmed Turki, Khaled El Matri, Leila El Matri, Daniel F. Schorderet

**Affiliations:** 1 IRO-Institute for Research in Ophthalmology, 1950 Sion, Switzerland; daniel.schorderet@irovision.ch; 2 Oculogenetic laboratory LR14SP01, Hedi Rais Institute of Ophthalmology (Department B), Tunis 1007, Tunisia; yosra.falfoul@yahoo.fr (Y.F.); ahmed.turki@web.de (A.T.); khaled.elmatri@gmail.com (K.E.M.); leilaelmatri@gmail.com (L.E.M.); 3 Department of Ophthalmology, Cantonal Hospital St. Gallen, 9000 St. Gallen, Switzerland; margaritatodorova@yahoo.com; 4 Department of Ophthalmology, University of Basel, 4000 Basel, Switzerland; 5 Eye Clinic, Lucerne Cantonal Hospital, 6000 Lucerne, Switzerland; stefan.wyrsch@hin.ch (S.W.); maria.helfenstein@luks.ch (M.H.); 6 Jules Gonin Eye Hospital, 1004 Lausanne, Switzerland; veronikavaclavik@yahoo.co.uk; 7 Department of Ophthalmology, University of Lausanne, 1004 Lausanne, Switzerland; 8 Faculty of Life Sciences, Ecole polytechnique fédérale de Lausanne, 1004 Lausanne, Switzerland

The authors wish to make a correction to the published version of their paper [1] because they misread a finding that they cited from the article by Chibani et al. [2] (This reference is cited as [9] in the original text.).

In the second paragraph of Section 4.2, the sentence “This alteration was not reported in the 1000 Genome Project or in the ExAC database and was only recently reported in compound heterozygous state [9].” should be changed to “This alteration was not reported in the 1000 Genome Project or in the ExAC database but has recently been found [9].”

The authors would like to apologize for any inconvenience caused. The change does not affect the scientific results. The manuscript will be updated and the original will remain online on the article webpage, with a reference to this correction.
